# Comment on “The Ocular Surface Chemical Burns”

**DOI:** 10.1155/2015/541512

**Published:** 2015-01-31

**Authors:** Abdullah Ilhan, Umit Yolcu

**Affiliations:** ^1^Ophthalmology Department, Erzurum Military Hospital, 25020 Erzurum, Turkey; ^2^Ophthalmology Department, Sarikamis Military Hospital, 36500 Kars, Turkey

We have read the paper entitled “The Ocular Surface Chemical Burns” by M. Eslani et al. with great interest [1]. We congratulate the authors that they composed a thorough review with precious pearls and would like to make a contribution.

In case of a chemical injury, if stromal nerve endings get seriously harmed, sensation and pain could be reduced. This would be in contradiction with the severity of the injury and might mislead the physician. Furthermore, while examining such a patient, physicians ought to avoid phenylephrine drops due to the increased risk of ischemia.

The authors warn us about lime injuries for the risk of ongoing damage if they are trapped in the deep fornixes. Meanwhile, there is a positive feature of lime injuries that their damage is restricted by the formation of calcium soaps that precipitate and prevent deeper penetration [2]. Another circumstance mentioned in the text is the combined injuries like exploding car batteries. Firework injuries also need to be handled cautiously because fireworks contain magnesium hydroxide which may cause a combined chemical and thermal injury.
